# Staged cranial resection of a left parietal high-grade glioma following intraoperative hemodynamic instability: a case report

**DOI:** 10.1093/jscr/rjag784

**Published:** 2026-09-04

**Authors:** Ana Itiel Jiménez-Ávila, Ricardo A Cortés-Monterrubio, Michelle A Macias-Grageda, Alexis E García-García, Coltzinc H Hernández-Jardón, Daniel A Vega-Moreno

**Affiliations:** Department of Neurosurgery, Centro Médico ISSEMYM-Toluca, Av. Baja Velocidad, 284 km, San Jeronimo Chicahualco, 52170, Mexico State, Mexico; Department of Neurosurgery, Centro Médico ISSEMYM-Toluca, Av. Baja Velocidad, 284 km, San Jeronimo Chicahualco, 52170, Mexico State, Mexico; Postgraduate Division, Neurosurgery Department, Universidad Nacional Autónoma de México, Escolar 411A, Copilco Universidad, 04360, Coyoacán Ciudad de México, México; Department of Neurosurgery, Centro Médico ISSEMYM-Toluca, Av. Baja Velocidad, 284 km, San Jeronimo Chicahualco, 52170, Mexico State, Mexico; Department of Neurosurgery, Centro Médico ISSEMYM-Toluca, Av. Baja Velocidad, 284 km, San Jeronimo Chicahualco, 52170, Mexico State, Mexico; Department of Neurosurgery, Centro Médico ISSEMYM-Toluca, Av. Baja Velocidad, 284 km, San Jeronimo Chicahualco, 52170, Mexico State, Mexico; Department of Neurosurgery, Centro Médico ISSEMYM-Toluca, Av. Baja Velocidad, 284 km, San Jeronimo Chicahualco, 52170, Mexico State, Mexico; Postgraduate Division, Neurosurgery Department, Universidad Nacional Autónoma de México, Escolar 411A, Copilco Universidad, 04360, Coyoacán Ciudad de México, México

**Keywords:** high-grade glioma, staged cranial surgery, intraoperative hemorrhage, hemodynamic instability, sodium fluorescein, neuronavigation

## Abstract

Unplanned staged resection for primary intra-axial tumors is rarely described in adults. A 54-year-old man with a left parietal high-grade glioma underwent decompressive craniectomy with attempted cytoreduction; an intraoperative hypertensive crisis triggered refractory venous hemorrhage and progressive cerebral edema, forcing the procedure to stop. He recovered neurologically (GCS 15) and returned 85 days later for a second-stage left parietal lobectomy using neuronavigation and sodium fluorescein-enhanced visualization, gross total resection, no complications. In high-grade glioma, hemodynamic instability need not preclude a complete resection; staging the procedure after physiological recovery appears to be a safe route when the initial operation cannot continue.

## Introduction

High-grade gliomas (HGGs), particularly IDH-wildtype glioblastoma (WHO grade 4), are the most aggressive primary brain tumors in adults. With optimal multimodal therapy (maximal safe resection, radiotherapy, and temozolomide), median overall survival ranges from 15 to 18 months [1]. Resection depth is one of the few modifiable prognostic factors: an extent of resection (EOR) above 98% of the contrast-enhancing tumor volume has been associated with improved outcomes in multiple series [2, 3].

Intraoperative hemorrhage is among the most serious complications of glioma surgery. Severe bleeding can force incomplete resection and, when hemostasis requires sacrifice of vascular structures, may precipitate ischemic stroke [4]. Pressing forward under those conditions often trades a marginal gain in EOR for a disproportionate increase in neurological risk, which is the practical argument for a staged resection.

Dandy first described staged cranial surgery in 1925 for large intracranial lesions in the context of skull base tumors [5]. For primary intra-axial neoplasms, the approach is rarely used, and unplanned staging driven specifically by intraoperative hemodynamic compromise in adults has appeared in only isolated reports.

We describe a patient with a left parietal HGG in whom the first stage was stopped because of a hypertensive crisis with refractory venous hemorrhage and progressive cerebral edema. After clinical stabilization, a second-stage lobectomy guided by neuronavigation and sodium fluorescein achieved a gross total resection without neurological deterioration.

## Case presentation

**Figure 1:**
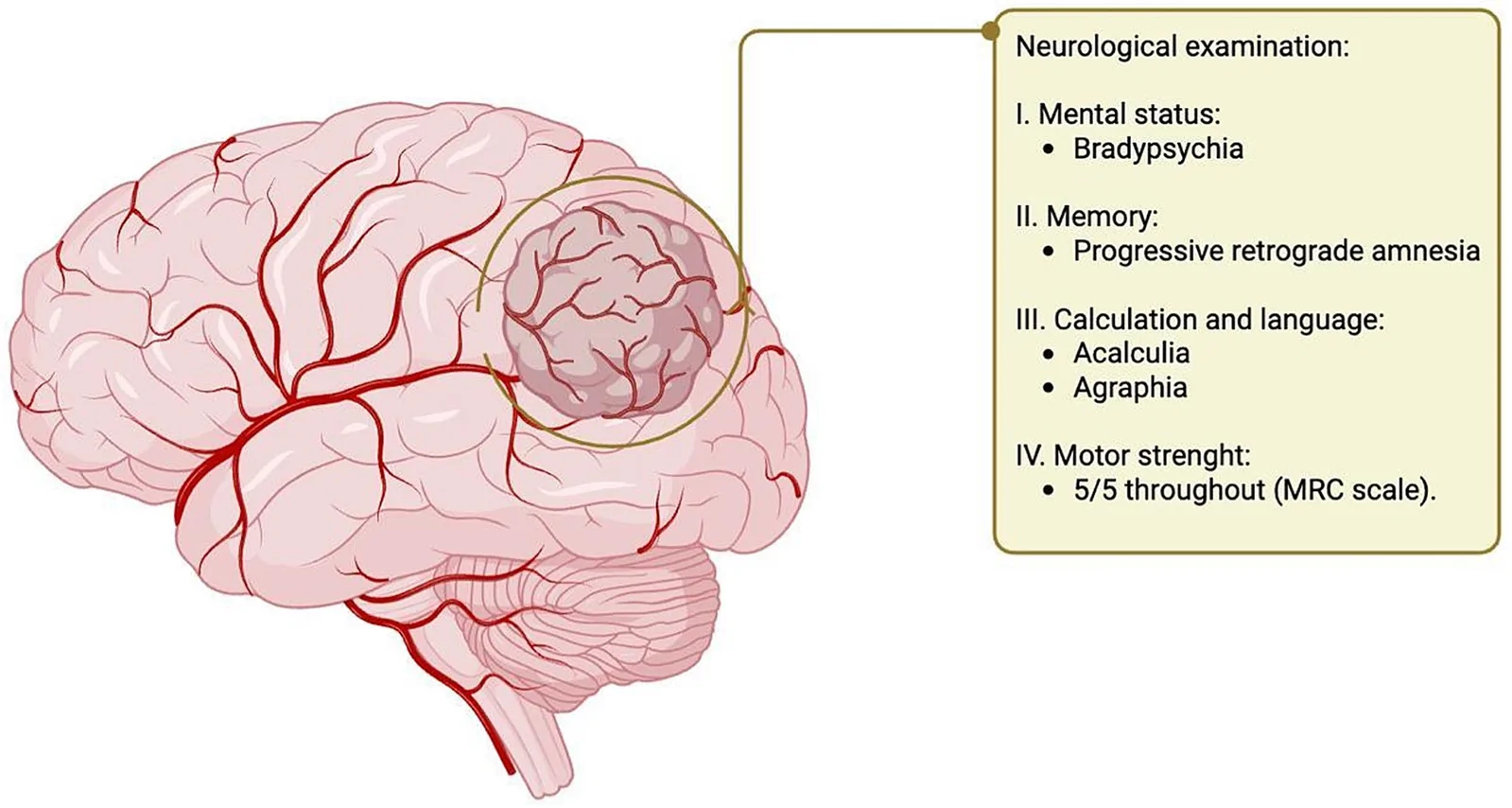
Neurological Assessment Schematic of the neurological examination in relation to the lesion, presenting an incomplete Gerstmann syndrome. Motor strength was 5/5 throughout (MRC scale).

**Figure 2:**
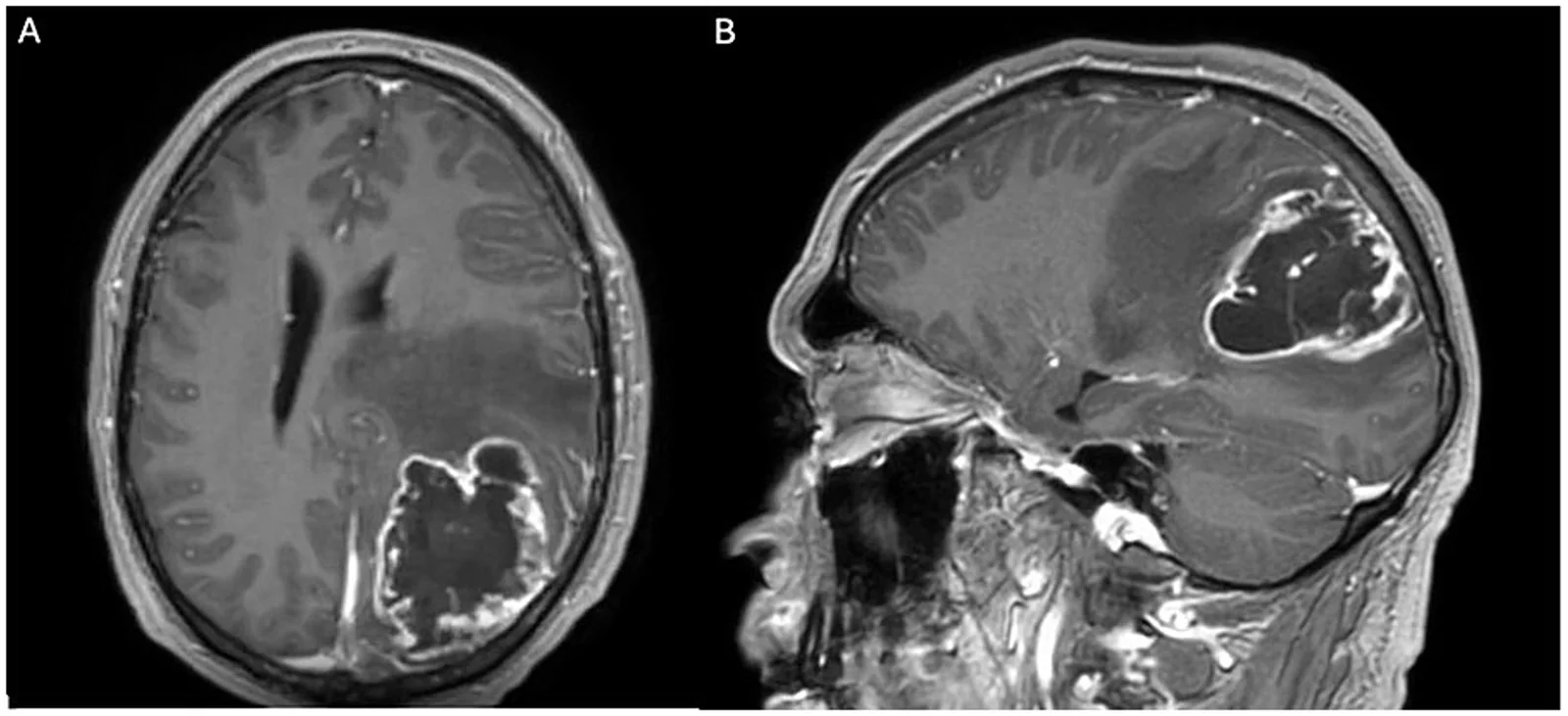
Preoperative neuroimaging T1 gadolinium-enhanced MRI. (A) Axial projection showing a left parietal intra-axial lesion with ring-like enhancement. (B) Sagittal projection revealing extensive perilesional vasogenic edema and a midline shift.

**Figure 3:**
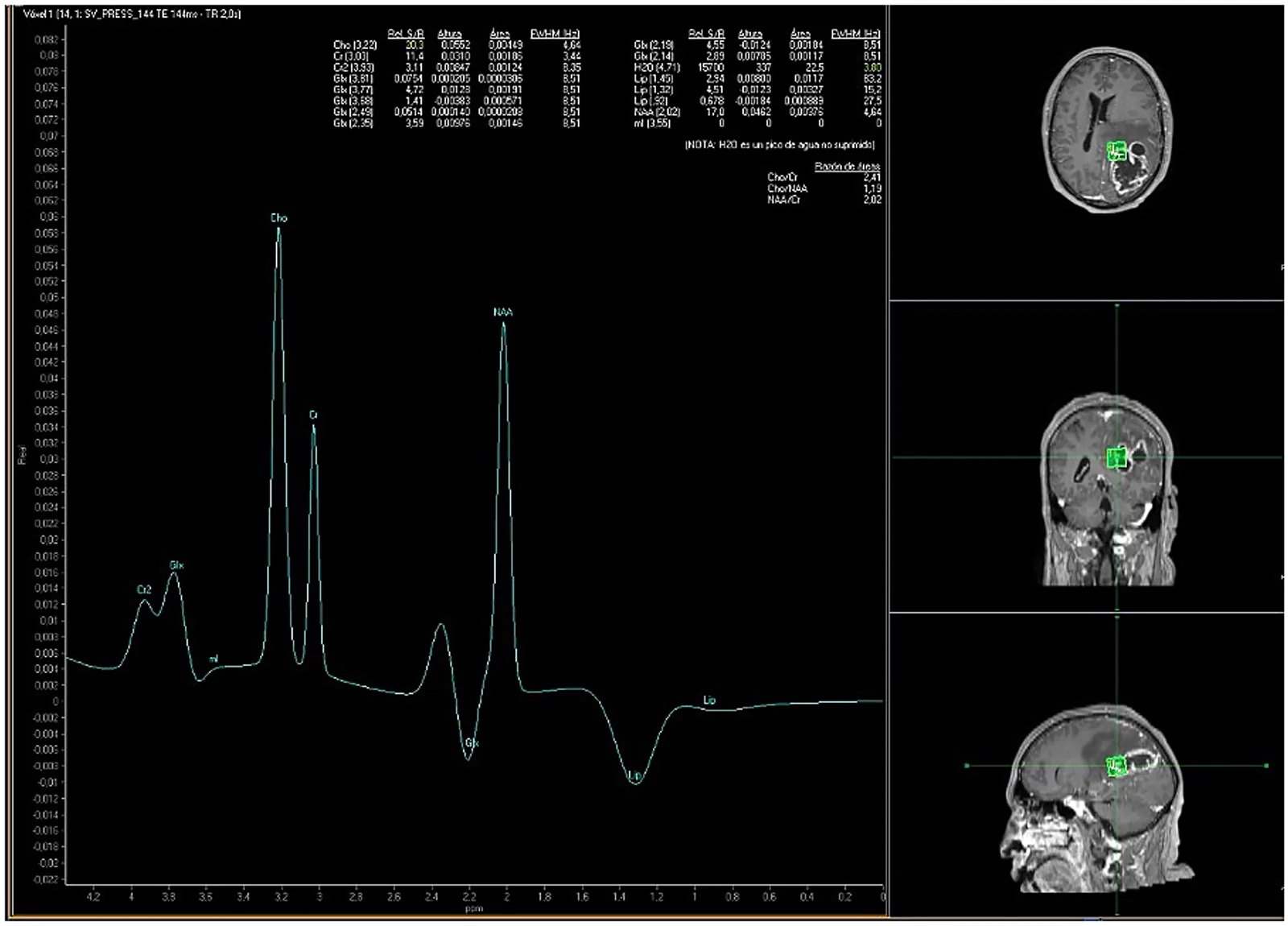
Proton MR spectroscopy analysis of the left parietal lesion showing significant peaks of *N*-acetylaspartate and choline, compatible with a high-grade tumor lesion.

A 54-year-old man with no relevant medical history presented in October 2023 with a two-month history of episodic vertigo and progressive retrograde amnesia. Neurological examination showed bradypsychia, acalculia, and agraphia consistent with an incomplete Gerstmann syndrome. Motor strength was 5/5 throughout (MRC scale) with no cranial nerve deficits or pyramidal signs (Fig. 1). Gadolinium-enhanced magnetic resonance imaging (MRI) on 27 December 2023, revealed a heterogeneous left parietal intra-axial lesion isointense on T1, with hypointense areas consistent with necrosis and cystic degeneration, poorly defined borders, extensive perilesional vasogenic edema, and a midline shift of 7.32 mm (Figs 2 and 3).

**Figure 4:**
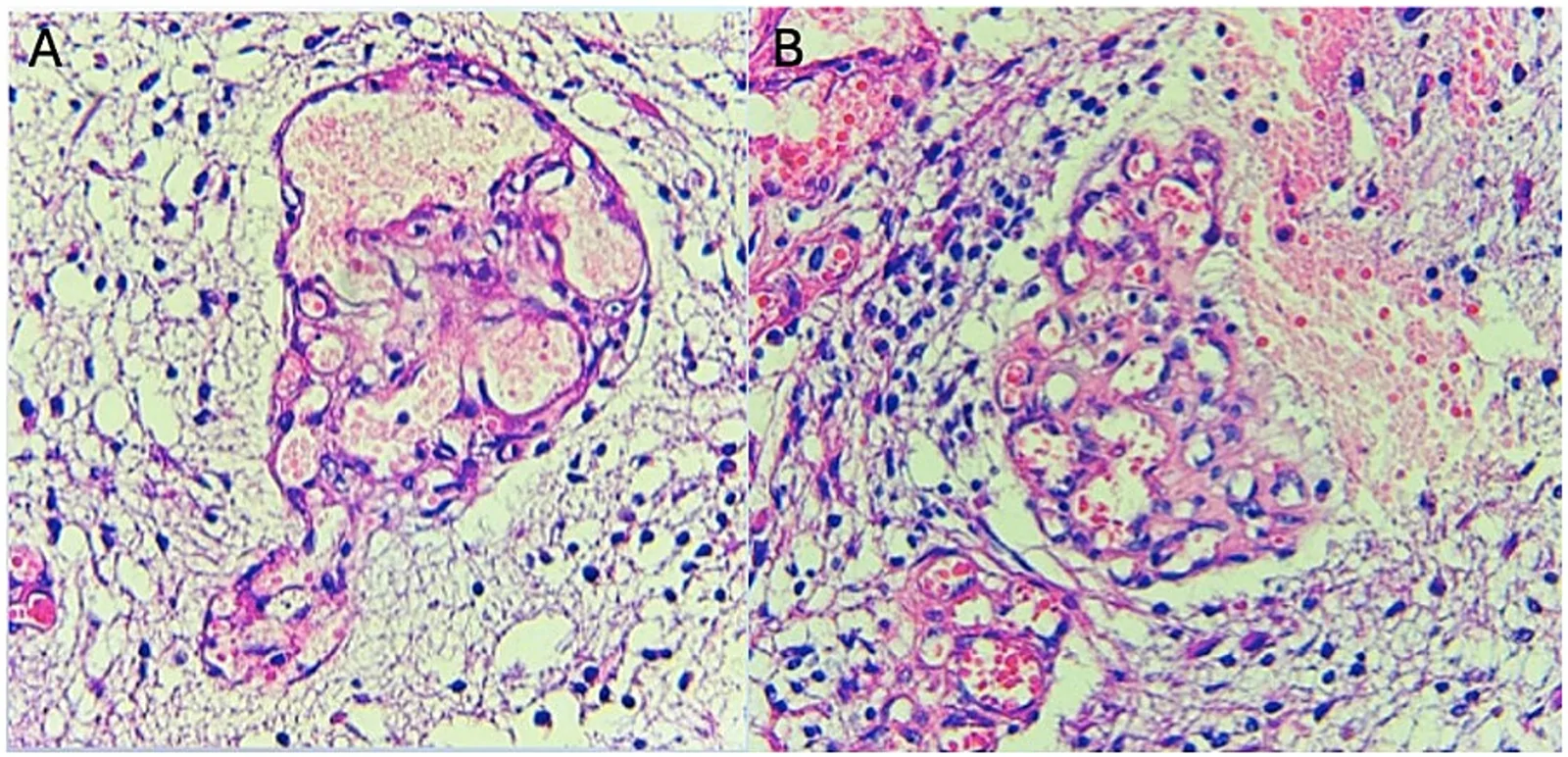
Histopathological Analysis. (a) Hematoxylin and eosin (H&E) stain showing areas of necrosis. (B) Slide demonstrating intravascular endothelial hyperplasia compatible with diffuse glioma of adult type, histologically grade 4.

**Figure 5:**
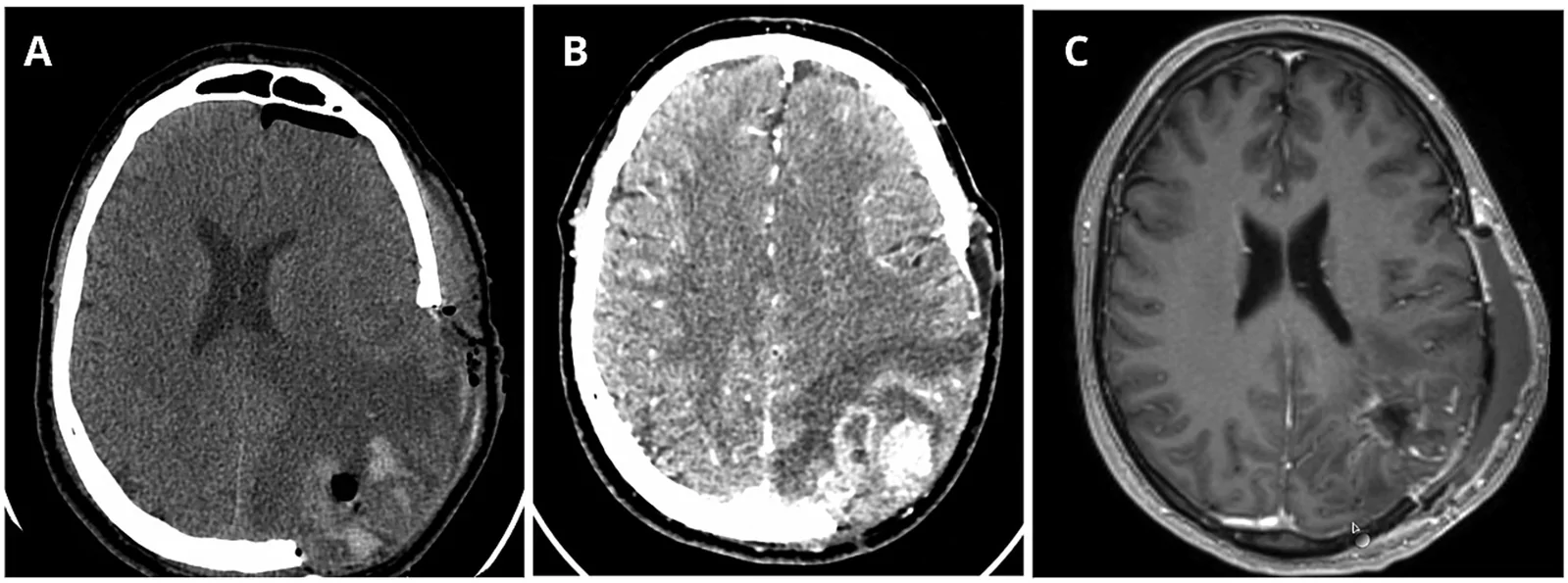
Post-surgical image stage 1. Pre-surgical image stage 2. Post-surgical image stage 2. (A) Postoperative image of stage 1 with evidence of partial resection, cerebral edema, intratumoral hemorrhage and transcalvarial hernia without evidence of cerebral infarction. (B) Preoperative image of the second phase showing residual lesion with contrast enhancement and cerebral edema. (C) Postoperative image from the second stage. Evidence of maximum safe resection, with no residual lesion that reacts to the contrast medium.

The patient underwent a left frontoparietal decompressive craniectomy with intended cytoreduction on 30 December 2023. An acute hypertensive crisis during resection produced spontaneous venous hemorrhage unresponsive to bipolar cauterization and hemostatic packing. Progressive cerebral edema persisted despite partial cytoreduction and fluid optimization. Given the sustained hemodynamic compromise and the risk of further neurovascular injury, the resection was aborted with residual tumor left *in situ*. Postoperative computed tomography showed no infarction, postsurgical hematoma, or herniation (Fig. 4). The patient required brief mechanical ventilation, was extubated without incident, and was discharged with GCS 15. Bradypsychia had improved. Acalculia and agraphia persisted. Histopathology confirmed high-grade glioma (Fig. 5).

Approximately 85 days later, the patient was readmitted with progressive increased volume at the craniectomy site and nausea. GCS remained 15 with unchanged deficits. Repeat neuroimaging showed residual tumor without interval growth, ischemia, or new hemorrhage. On 25 March 2024, a left parietal lobectomy was performed through the prior craniectomy. Neuronavigation and sodium fluorescein-enhanced visualization were used to delineate tumor margins within the altered operative field. Gross total resection was achieved without intraoperative incidents. The patient was discharged on postoperative Day 2 at GCS 15. Agraphia had improved and mild acalculia remained. No surgical, infectious, or thromboembolic complications occurred.

## Discussion

The intraoperative decision to abort the resection was driven by a hypertensive crisis, uncontrolled venous hemorrhage, and progressive cerebral edema. Continuing surgery under these conditions would have significantly increased the risk of permanent neurological deficit or death.

A second surgical procedure was decided upon, due to the residual volume of the lesion, as well as the clinical worsening presented by the patient, based on the fact that maximum safe resection associated with complementary medical treatment improves the survival of patients with high-grade glioma, therefore despite the complications that occurred in the first surgical procedure the patient underwent a reintervention.

HGGs exhibit abnormal vascular architecture due to dysregulated angiogenesis and endothelial proliferation, predisposing to intraoperative bleeding [4]. Postoperative hemorrhage occurs in up to 10.8% of cases, with reoperation required in 1.3%–3% [6, 7]. Elevated perioperative systolic blood pressure (>160 mmHg) has been identified as a risk factor for hematoma formation [8], emphasizing the importance of strict hemodynamic control.

Staged resection offers a pragmatic solution when intraoperative conditions become unfavorable. Although rarely reported, available evidence suggests that staged procedures do not increase complication rates compared to single-stage surgery [5]. In this case, staging allowed physiological recovery and safe completion of resection.

The 85-day interval falls within reported ranges but represents the upper limit. While shorter intervals are preferable given the aggressive nature of HGGs, no tumor progression was observed, allowing successful resection. This delay was influenced by logistical factors and should not be generalized.

Neuronavigation combined with sodium fluorescein visualization was particularly useful during the second procedure. Prior surgery distorts anatomical landmarks and tissue planes, which limits the reliability of neuronavigation when used alone. Sodium fluorescein accumulates where the blood–brain barrier is disrupted (as it is in HGGs) and provides real-time delineation of residual tumor [9]. Using both adjuncts together offered anatomical orientation and a tumor-specific optical signal, which made gross total resection feasible in an operative field substantially altered by the prior procedure and residual edema.

Both stages were tolerated without permanent neurological deterioration. The patient returned to GCS 15 after each procedure, with progressive improvement of agraphia and stable mild acalculia, an expected outcome for a lesion in the dominant parietal lobe. No motor deficits emerged and no perioperative complications were recorded after the second stage. These results support the view that staged resection, when appropriately indicated, can reduce the morbidity that would otherwise result from forcing completion of a high-risk procedure under adverse physiologic conditions.

## Conclusion

Staged cranial resection for primary intra-axial HGGs is exceedingly rare in adults, and unplanned staging driven by intraoperative hemodynamic instability is even less frequently reported. Early surgical termination in the setting of refractory venous hemorrhage and cerebral edema is justified when continuation would carry unacceptable neurological risk. After adequate physiologic recovery, second-stage resection with multimodal intraoperative guidance can achieve gross total tumor removal with preservation of function. Prospective studies are needed to define optimal inter-stage timing, patient selection criteria, and the long-term oncological implications of this approach.

## Data Availability

All data supporting the findings of this study are contained within the article.

## References

[1] Stupp R, Mason WP, van den Bent MJ et al. Radiotherapy plus concomitant and adjuvant temozolomide for glioblastoma. N Engl J Med 2005;352:987–96. 10.1056/NEJMoa043330 15758009

[2] Della Pepa GM, Ius T, Menna G et al. Maximal safe resection in glioblastoma surgery: a systematic review of advanced intraoperative image-guided techniques. Brain Sci 2023;13:216. 10.3390/brainsci13020216 36831759 PMC9954589

[3] Lacroix M, Abi-Said D, Fourney DR et al. A multivariate analysis of 416 patients with glioblastoma multiforme: prognosis, extent of resection, and survival. J Neurosurg 2001;95:190–8. 10.3171/jns.2001.95.2.0190 11780887

[4] Welling LC, Akinduro OO, Catapano JS et al. Handling severe intraoperative hemorrhage and avoiding iatrogenic stroke during brain tumor surgery. World Neurosurg 2023;178:1–12. 37178915 10.1016/j.wneu.2023.05.019

[5] Bui N, Brown N, Dzihic E et al. Staged open cranial surgery for primary intra-axial neoplasms: a systematic review. World Neurosurg 2023;175:e167–73. 10.1016/j.wneu.2023.03.046 36924887

[6] Lenga P, Kleineidam H, Trong P et al. Optimizing patient outcome in intracranial tumor surgery: a detailed prospective study of adverse events and mortality reduction strategies. Acta Neurochir 2024;166:126. 10.1007/s00701-024-06008-y 38457057 PMC10923735

[7] Sajjad J, Kaliaperumal C, Yousaf I et al. A prospective analysis of complications of intracranial tumor surgery. J Neurol Surg A Cent Eur Neurosurg 2017;78:53–9. 27415593 10.1055/s-0036-1584814

[8] Chen L, Li Y, Sun Y et al. Early unplanned reoperation after glioma craniotomy: incidence, predictors and process improvement. Front Oncol 2022;12:1–7. 10.3389/fonc.2022.898873 PMC9121807 35600362

[9] Acerbi F, Broggi M, Schebesch KM et al. Fluorescein-guided surgery for resection of high-grade gliomas: a multicentric prospective phase II study (FLUOGLIO). Clin Cancer Res 2018;24:52–61. 10.1158/1078-0432.CCR-17-1184 29018053

